# Comorbidity amplifies the effects of post-9/11 posttraumatic stress disorder trajectories on health-related quality of life

**DOI:** 10.1007/s11136-017-1764-5

**Published:** 2017-12-20

**Authors:** Jiehui Li, Kimberly Caramanica Zweig, Robert M. Brackbill, Mark R. Farfel, James E. Cone

**Affiliations:** New York City Department of Health and Mental Hygiene, World Trade Center Health Registry, 125 Worth Street, CN-6W, New York, NY 10013 USA

**Keywords:** PTSD, Health-related quality of life, Comorbidity, World Trade Center

## Abstract

**Purpose:**

The present study aims to examine the impact of physical and mental health comorbidities on the association between post-9/11 posttraumatic stress disorder (PTSD) trajectories over 10 years and health-related quality of life (HRQOL) among 9/11-exposed persons.

**Methods:**

30,002 responding adult World Trade Center Health Registry enrollees reporting no pre-9/11 PTSD were studied. PTSD trajectories (chronic, delayed, remitted, no PTSD) were defined based on a 17-item PTSD Checklist-Specific to 9/11 across three waves of survey data. Three indicators of poor HRQOL were defined based on CDC HRQOL-4 measures. We computed age-adjusted prevalence of physical and mental health comorbidity (depression/anxiety) by PTSD trajectory and used modified Poisson regression to assess the effect of PTSD trajectory on poor HRQOL prevalence, accounting for comorbidity.

**Results:**

Age-adjusted prevalence of overall comorbid conditions was 95.8 and 61.4% among the chronic and no-PTSD groups, respectively. Associations between 9/11-related PTSD trajectories and poor HRQOL were significant and became greater when comorbidity was included. Adjusted prevalence ratios were elevated for fair/poor health status (APR 7.3, 95% CI 6.5, 8.2), ≥ 14 unhealthy days (4.7; 95% CI 4.4, 5.1), and ≥ 14 activity limitation days during the last 30 days (9.6; 95% CI 8.1, 11.4) in the chronic PTSD group with physical and mental health comorbidity compared to those without PTSD and comorbidity; similar associations were observed for delayed PTSD.

**Conclusions:**

Ten years post-9/11 physical and mental health comorbidities have a substantial impact on the PTSD trajectories and HRQOL association. The need for early identification and treatment of PTSD and comorbidity should be emphasized to potentially improve HRQOL.

**Electronic supplementary material:**

The online version of this article (10.1007/s11136-017-1764-5) contains supplementary material, which is available to authorized users.

## Introduction

The September 11, 2001 (9/11) terrorist attacks on the World Trade Center (WTC) in New York City (NYC) exposed hundreds of thousands of individuals to an immense cloud of dust and debris, numerous hazardous substances, and emotional trauma. Those with these experiences have an increased risk of mental disorders and comorbid mental and physical health conditions. To prevent disability and optimize functioning and quality of life among those impacted by 9/11 exposure, it is important to understand the burden of mental and physical conditions among the exposed population and to address the well-being of individuals with mental and/or physical health conditions long after the exposure.

Posttraumatic stress disorder (PTSD) is one of the most commonly reported mental health conditions following exposure to the 9/11 disaster [1–6]. PTSD symptoms can last for many years [7]. Chronic PTSD symptoms among 9/11-exposed responders and civilians over 8–9 years of follow-up have been reported [8–10]. Pietrzak et al. reported that 5.3% of police and 9.5% of non-traditional responders experienced chronic PTSD, while 8.5% of police and 6.7% of non-traditional responders reported delayed-onset PTSD [8]. The WTC Health Registry reported similar findings: 4.0% chronic PTSD and 6.4% delayed-onset PTSD among rescue/recovery workers [9] and 8.0% chronic and 8.2% delayed-onset PTSD among civilians not involved in rescue/recovery work [10]. The effects of chronic post-9/11 PTSD on quality of life can be substantial due to long-lasting or re-occurring symptoms and increased risk of comorbid health conditions.

PTSD is often comorbid with other psychological disorders [11–13], as well as physical health conditions. Mental disorders of all kinds are associated with increasing risk of subsequent onset or diagnosis of a number of physical conditions, such as heart and lung diseases, diabetes, and arthritis [14]. The rates and burden of comorbidity are high in those with 9/11-related PTSD [5, 15–18]. The prevalence of depression among 9/11 responders with PTSD ranged from 66.5% [5] to 73.0% [15, 16]. The prevalence of anxiety among police 9/11 responders with PTSD was 53.5% [13]. Among responders with PTSD, 70.0% reported at least one of three aerodigestive disorders (asthma, sinusitis, and gastroesophageal reflux disease) [16]. Among civilian survivors, 40% of those with PTSD reported lower respiratory symptoms [17]. Nevertheless, the burden of comorbid physical health conditions in persons with 9/11-related PTSD may be greater if accounting for a wider range of physical health conditions beyond aerodigestive disorders [16–18].

When evaluating the burden of diseases, there has been increased focus on measuring the patient’s perspective. Health-related quality of life (HRQOL) is a measure of an individual’s perceived physical and mental health over time, and an indicator of disease burden. The adverse effects of PTSD [19], depression [20], anxiety [21], and physical health conditions [22] on HRQOL have been well documented in various populations. PTSD has been associated with reduced HRQOL [19], and may result in worse HRQOL in the presence of comorbid depression, anxiety, or physical health conditions [23, 24]. Individuals with PTSD and depression have worse QOL than those with PTSD alone [23]. There is evidence suggesting PTSD and depression both independently contributed to mental HRQOL [23, 25]. A population-based study of war veterans found that those who screened positive for PTSD reported a mean of 20 physical health symptoms compared with a mean of four symptoms among those without PTSD [24]. In the same study, 58% of those with PTSD rated their general health status as poor or fair while only 6% of those without PTSD rated health status as poor or fair [24]. Persistent PTSD symptoms measured at two discrete time points over a 14-year period among Vietnam veterans were found to be associated with worse family relationships, less life satisfaction and happiness, more mental health service use, and more non-specific health complaints than those without [26]. PTSD often has a duration of many years [7], and is characterized by frequent comorbid mental and physical health conditions among 9/11 exposed populations [5, 13, 15–18]. It is essential to understand how the long-term course of 9/11-related PTSD 10 years post-disaster influences HRQOL based on more frequent follow-up data.

The 9/11 exposed individuals with comorbid physical and mental health conditions have worse HRQOL than those without PTSD or with PTSD alone years after the event [5, 17, 18]. A recent study of 9/11 responders reported that persons with active PTSD were 2.6 times more likely and those with remitted PTSD 1.8 times more likely than unaffected responders to report having fair/poor overall health status after adjusting for depression and other covariates [6]. In other previous studies of 9/11-exposed populations, those with PTSD and depression, or those with PTSD and lower respiratory symptoms were more than two times as likely as patients with PTSD alone to report ≥ 14 unhealthy days in the last 30 days [5, 17]. However, research examining the extent to which comorbid depression, anxiety, and chronic physical conditions affect the association between PTSD trajectory and HRQOL has been limited.

This report addresses two objectives: (1) to estimate the burden of medical morbidity by 9/11-related PTSD trajectory group a decade after 9/11; and (2) to estimate the impact of physical and mental health comorbidities on the association between 9/11-related PTSD trajectories and HRQOL. In this study we hypothesized that the prevalence of comorbid depression and/or anxiety, as well as comorbid physical health conditions would be higher among those who had more years of PTSD symptoms than those who had fewer years of symptoms. Individuals with PTSD who have comorbid psychological and physical health conditions would have significantly worse quality of life 10 years after the 9/11 disaster than those without comorbidity.

## Methods

### Study sample

Data came from the WTC Health Registry (“Registry”), a longitudinal closed cohort study created in 2002 as a public health response to 9/11 [27]. A total of 71,431 individuals who lived, worked, or went to school in the area of the WTC disaster on 9/11, or were involved in rescue and recovery work were enrolled on a voluntary basis in 2003–2004 (Wave 1, W1) [27]. Enrollees were either identified through lists provided by employers, government agencies, and other entities (30%), or they responded to an outreach campaign (70%). This study used data from W1 and two follow-up surveys conducted in 2006–2007 (Wave 2, W2) and 2011–2012 (Wave 3, W3). Of the 68,672 people aged ≥ 18 years on 9/11/2001 who enrolled in the Registry (W1), 35,857 (52.5%) participated in the next two follow-up surveys (W2 and W3). After excluding proxy responses (*n* = 309), enrollees reporting physician-diagnosed pre-9/11 PTSD (*n* = 497), enrollees missing items on the self-reported PTSD Checklist (PCL) assessment at any wave, or missing values on HRQOL measures, depression, and anxiety assessments at W3 (*n* = 5049), the final sample was 30,002.

The Centers for Disease Control and Prevention (CDC) and the NYC Department of Health and Mental Hygiene institutional review boards approved the World Trade Center Health Registry protocol.

### Measures

#### Health-related quality of life (HRQOL)

HRQOL, the outcome of interest, was assessed at W3 using the CDC HRQOL 4-item Healthy Days Measure (CDC HRQOL-4) [28, 29]. The CDC HRQOL-4 assesses an individual’s perceived sense of well-being through four questions: (1) Would you say that in general your health is excellent, very good, good, fair, or poor?; (2) Thinking about your physical health, which includes physical illness and injury, for how many days during the last 30 days was your physical health not good?; (3) Thinking about your mental health, which includes stress, depression, and problems with emotions, for how many days during the last 30 days was your mental health not good?; and (4) For about how many days did poor physical or mental health keep you from doing your usual activities during the last 30 days? These HRQOL measures have been shown to have good validity and reliability [28, 29].

Based on response to above questions, three indicators of poor HRQOL were derived for use in this study to assess different aspects of HRQOL [28]: (1) general health status, dichotomized into poor or fair vs. good, very good, or excellent, to signify overall self-rated health; (2) unhealthy days (the sum of physical and mental unhealthy days), with a logical maximum of 30 unhealthy days (Centers for Disease Control and Prevention) (≥ 14 vs. < 14 days), to measure recent physical symptoms and/or mental distress ; and (3) activity limitation days (≥ 14 vs. < 14 days), to indicate perceived disability as well as lower productivity [28, 29].

#### Post-9/11 PTSD trajectory

Post-9/11 probable PTSD trajectory was the main predictor of interest. Probable PTSD was assessed at each wave using the PTSD Checklist-Specific (PCL-S), a 17-item self-reported symptom scale, which referred specifically to the events of 9/11. The 17 PCL-S items correspond to the three PTSD symptom clusters (re-experiencing, avoidance, hyperarousal) from the Diagnostic and Statistical Manual of Mental Disorders (DSM-IV) [30]. The PCL-S is a well-validated measure and has good temporal stability, internal consistency (*α* > 0.75), test–retest reliability (correlation coefficient, *r* = 0.66), and high convergent validity (*r* = 0.58–0.93) [31]. Enrollees were asked to rate the degree to which they were bothered by symptoms in the past 30 days from 1 (not at all) to 5 (extremely). Responses to the 17 items were summed, for a total score of 17 to 85. Probable PTSD (subsequently referred to as PTSD) was defined as a PCL-S score ≥ 44 (overall diagnostic efficiency = 0.90, sensitivity = 0.94, and specificity = 0.86) [32], with at least one re-experiencing symptom (DSM-IV criterion B), three avoidance symptoms (DSM-IV criterion C), and two hyperarousal symptoms (DSM-IV criterion D) [33]. In the present sample, the internal consistency was excellent (Cronbach’s alpha = 0.95). Enrollees were further categorized into four trajectory groups based on their PTSD status across waves: (1) chronic (W1+ and W3+); (2) delayed (W1− and W3+); (3) remitted ((W1+ or W2+) and W3−); and (4) no PTSD (W1− and W2− and W3−).

#### Other covariates

The main confounders included in multivariate analyses were mental health (depression and/or anxiety, referred to as depression/anxiety) and physical health comorbidity. Current depression was measured at W3 using the 8-item Patient Health Questionnaire (PHQ-8) and defined as a PHQ-8 score ≥ 10 (positive predictive value = 96.5%) [34]. The PHQ-8 consists of eight out of nine criteria on which a DSM-IV depression diagnosis is based [30]. Anxiety was measured at W3 using the 7-item Generalized Anxiety Disorder Scale (GAD-7) and defined as a GAD-7 score ≥ 10 (internal consistency, *α* = 0.92; intra-class correlation = 0.83) [35]. Both the PHQ-8 and GAD-7 ask enrollees to rate how often they were bothered by a symptom during the last 2 weeks on a 4-point scale from 0 (not at all) to 3 (nearly every day).

Physical health comorbidity was defined based on self-report of any of 18 physician-diagnosed medical conditions at W3. These conditions included respiratory problems (asthma, reactive airways dysfunction syndrome (RADS), emphysema, chronic bronchitis, pulmonary fibrosis, or asbestosis), cardiovascular problems (hypertension, heart attack, angina, coronary heart disease, or stroke), autoimmune and endocrine problems (diabetes, rheumatoid arthritis, multiple sclerosis, amyotrophic lateral sclerosis, lupus, scleroderma, polymyositis, or thyroid), gastroesophageal reflux disease, and sarcoidosis.

Other covariates included in the multivariate analyses were sociodemographic characteristics, Registry enrollee eligibility group (rescue/recovery workers and volunteers vs. community members), and social support. Sociodemographic characteristics included age, gender, race/ethnicity, body mass index (BMI), physical activity, smoking history (current, former, never), household income (< $75,000 vs. ≥ $75,000), and employment status (unable to work due to poor health, retired, unemployed/other, employed). A social support measure covering multiple dimensions (e.g., emotional and tangible support) was assessed at W3. Respondents rated five items, including whether someone was available when needed to take them to the doctor, have a good time with, hug them, prepare meals, and understand their problems using a five-point scale from none (0) to all (4) of the time. The five items were summed, with a total score ranging from 0 to 20. The total score was then used to create a four-level social support variable categorized as none or little (0–5), some (6–10), most (11–15), or all (16–20) of the time.

### Statistical analysis

We first described the study population using frequency and proportions. We then computed age-adjusted (2000 U.S. Standard Population) prevalence of overall and individual comorbidity for all studied individuals and by 4-level PTSD trajectory using SAS-callable SUDAAN. Overall comorbidity was defined based on the presence of any depression or anxiety and any of the physical health conditions and categorized into 4 groups: comorbid physical and mental conditions, comorbid depression and/or anxiety alone, any comorbid physical condition alone, and no comorbidity. To examine the association between PTSD trajectory and each of the three indicators of poor HRQOL, we used modified Poisson regression with a robust error variance [36] to estimate the prevalence ratios directly. In multivariate analyses, we assessed the independent association between PTSD trajectory and each of the HRQOL indicators, respectively, in two separate analyses. Analysis 1 did not include physical and mental health comorbidity, and Analysis 2 included comorbidity, both analyses adjusted for sociodemographic characteristics, Registry eligibility group, and social support. In Analysis 2, comorbidity was represented as a 16-level categorical independent variable which combined PTSD trajectory and overall comorbidity status; the no-PTSD group with no comorbidity was the referent.

### Sensitivity analysis

Knowing that W3 non-participants were more likely to be younger, less educated, to have lower household income, and higher prevalence of 9/11-related PTSD compared to W3 participants [37], we conducted a sensitivity analysis to explore the potential impact of selection bias resulting from loss to follow-up. Specifically, we repeated Analysis 2 among W3 non-participants who participated in W2 and met similar inclusion criteria such as age ≥ 18 years on 9/11 and no pre-9/11 PTSD (Supplementary Table 1). Due to lack of PHQ-8 and GAD-7 data at W2, we instead used self-reported physician-diagnosed depression and anxiety. Additionally, information on some physical health conditions and social support was not available for the sensitivity analysis.

All analyses were performed in SAS Version 9.4 (SAS Institute, Inc., Cary, NC). Statistical significance was set at a two-sided 0.05 alpha level.

## Results

Table 1 describes the sociodemographic characteristics of the study sample (*n* = 30,002) at W3. The study enrollees were predominantly 45–64 years old (61.6%), male (63.0%), non-Hispanic white (74.2%), employed (69.0%), never smokers (56.1%), physically active (75.4%), overweight or obese (70.7%), community members (51.7%), had an annual household income ≥ $75,000 (60.4%), and frequent social support (76.4%). Most (65.6%) enrollees reported at least one of the 18 physical health conditions, and 19.0% had depression or anxiety at W3.

**Table 1:** Characteristics of study population at Wave 3 (*N* = 30,002)

	*N*	(%)^a^
Age, years		
25–44	7671	(25.6)
45–64	18,469	(61.6)
≥ 65	3862	(12.9)
Gender		
Male	18,892	(63.0)
Female	11,110	(37.0)
Race/ethnicity		
Non-Hispanic white	22,263	(74.2)
Non-Hispanic black	2589	(8.6)
Hispanic	2996	(10.0)
Asian and others	2154	(7.2)
Annual household income in 2010, $		
<75,000	10,538	(35.1)
≥75,000	18,132	(60.4)
Employment status		
Unable to work due to health	1834	(6.1)
Retired	5296	(17.7)
Unemployed and other	2072	(6.9)
Employed	20,710	(69.0)
Smoking status		
Current	3012	(10.0)
Former	10,081	(33.6)
Never	16,830	(56.1)
Physical inactivity during last month		
Yes	7229	(24.1)
No	22,633	(75.4)
Body mass index (BMI)		
≥30 (Obese)	9562	(31.9)
25 to < 30 (Overweight)	11,632	(38.8)
<25 (Normal/underweight)	8396	(28.0)
Perceived social support		
None-to-little	2565	(8.5)
Some of the time	4477	(14.9)
Most of the time	8466	(28.2)
All of the time	14,457	(48.2)
Registry enrollees		
Rescue/recovery workers	14,488	(48.3)
Community members	15,514	(51.7)
PTSD trajectory		
Chronic	1749	(5.8)
Delayed	2139	(7.1)
Remitted	2901	(9.7)
No PTSD	23,213	(77.7)
Ever reported physical health condition	19,691	(65.6)
Depression or anxiety	5709	(19.0)

*PTSD* posttraumatic stress disorder

^a^May not sum to 100% due to missing values

**Fig. 1:**
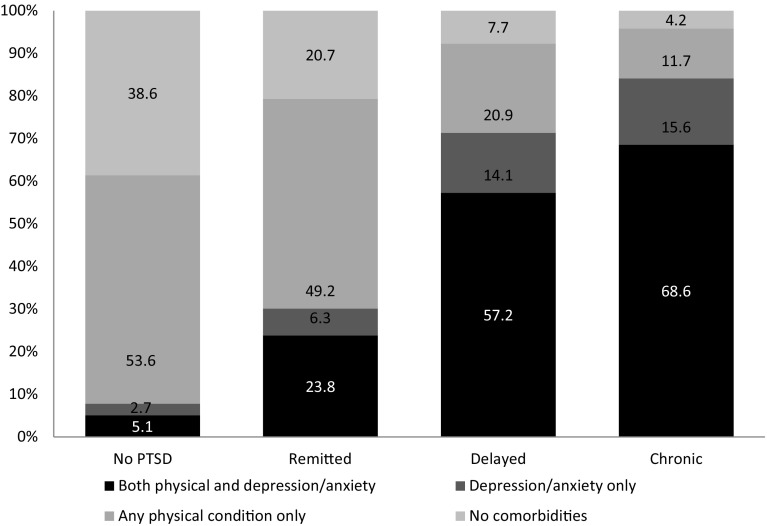
Age-adjusted prevalence of overall comorbidity status by PTSD trajectory

Age-adjusted prevalence of overall comorbidity by PTSD trajectory is depicted in Fig. 1. The age-adjusted prevalence of comorbid physical and mental health conditions was highest in the chronic PTSD (68.6%), followed by the delayed (57.2%), remitted (23.8%), and no-PTSD groups (5.1%). In contrast, the age-adjusted prevalence of no comorbidity was highest in the no-PTSD group (38.6%), and lowest in the chronic PTSD group (4.2%).

Age-adjusted prevalence of individual comorbidity in the study sample is shown in Table 2. The age-adjusted prevalence was 14.9% for depression and 12.1% for anxiety. Hypertension (32.3%), gastroesophageal reflux disease (21.4%), and asthma (19.6%) were the physical conditions with the highest age-adjusted prevalence. When stratified by PTSD trajectory, the prevalence of each of the mental or physical health conditions listed except for coronary heart disease and heart attack was highest for the chronic PTSD, followed by delayed PTSD, remitted PTSD, and the lowest for the no-PTSD group. The prevalence ratio of chronic to no-PTSD groups ranged from 1.4 for hypertension to 15.1 for anxiety (Table 2).

**Table 2:** Age-adjusted prevalence of physical and mental health comorbidity by PTSD trajectory (*N* = 30,002)^a^

	All	PTSD trajectory				Prevalence ratio (chronic/no PTSD)
		Chronic	Delayed	Remitted	No PTSD	
Mental health condition at Wave 3						
Depression (PHQ-8 score ≥ 10)	14.9	77.2	64.0	23.4	5.5	14.0
Anxiety (GAD-7 score ≥ 10)	12.1	66.4	50.9	18.3	4.4	15.1
Ever reported physical health condition						
Gastroesophageal reflux disease	21.4	39.4	36.0	27.0	18.2	2.2
Sarcoidosis	1.0	1.9	1.8	1.4	0.9	2.1
Respiratory problem						
Asbestosis	0.6	2.0	1.6	0.6	0.4	5.0
Asthma	19.6	35.0	29.8	26.7	16.9	2.1
Chronic bronchitis	8.7	23.0	20.7	13.9	6.2	3.7
Emphysema	4.2	10.8	10.4	5.5	3.1	3.5
Pulmonary fibrosis	0.8	3.4	2.0	1.4	0.5	6.8
Reactive airway dysfunction syndrome or RADS	3.6	10.7	9.5	5.1	2.4	4.5
Cardiovascular problem						
Angina	2.4	6.4	4.2	3.0	1.9	3.4
Coronary heart disease	4.6	7.3	7.8	5.0	4.1	1.8
Heart attack	2.7	4.6	4.9	3.4	2.4	1.9
Hypertension	32.3	43.7	41.4	37.1	30.2	1.4
Stroke	1.5	3.0	2.5	1.9	1.3	2.3
Autoimmune and endocrine disorders						
Diabetes	8.7	15.6	11.9	11.9	7.6	2.1
Multiple sclerosis or amyotrophic lateral sclerosis	0.6	0.8	0.7	0.7	0.5	1.6
Rheumatoid arthritis	6.0	15.1	10.7	9.5	4.6	3.3
Other autoimmune disorders (e.g., lupus, scleroderma, polymyositis)	2.7	6.0	4.3	3.4	2.3	2.6
Thyroid disease	7.9	12.1	10.8	9.5	7.2	1.7

*PTSD* posttraumatic stress disorder

^a^They are not mutually exclusive

Overall, 23.5% of enrollees reported having poor or fair health status, 32.3% reported ≥ 14 unhealthy days, and 11.9% reported ≥ 14 activity limitation days at W3 (data not shown). The prevalence of each indicator of poor HRQOL was highest among those with chronic PTSD (67.4% poor or fair health status, 83.5% ≥ 14 unhealthy days, and 48.5% ≥ 14 activity limitation days) and lowest among those with no PTSD (15.3% poor or fair health status, 22.3% ≥ 14 unhealthy days, and 6.3% ≥ 14 activity limitation days). When stratified by overall comorbidity status, the prevalence of each of the three indicators of poor HRQOL decreased as PTSD improved. For each PTSD trajectory group, those with physical and mental health comorbidity were most likely to report poor HRQOL and those with no comorbidities were least likely.

In multivariate analyses, adjusting for socio-demographics, Registry eligibility group, and social support (Table 3), PTSD trajectory was independently associated with each of the three indicators of poor HRQOL. In Analysis 1 without accounting for comorbidity, the adjusted prevalence ratio (APR) for each indicator of poor HRQOL was truncated but remained significant, with an APR ranging from 1.8 to 3.3. Individuals with chronic PTSD had elevated risk of poor or fair health status (APR 2.2; 95% CI 2.1, 2.3), ≥ 14 unhealthy days (APR 2.3; 95% CI 2.2, 2.4), and ≥ 14 activity limitation days (APR 3.3; 95% CI 3.1, 3.7) compared to the no-PTSD group. Similar associations were observed for delayed PTSD.

**Table 3:** Adjusted prevalence ratio (APR) of PTSD trajectory with poor HRQOL by physical and mental comorbidity among adult WTCHR enrollees (*N* = 30,002)

	*N*	APR (95% confidence interval)^a^		
		Fair/poor general health	≥ 14 Unhealthy days^b^	≥ 14 Activity limitation days^b^
Analysis 1^c^				
Probable PTSD at Wave 3				
Chronic	1749	2.2 (2.1–2.3)	2.3 (2.2–2.4)	3.3 (3.1–3.7)
Delayed	2139	2.2 (2.1–2.3)	2.3 (2.3–2.5)	3.1 (2.9–3.4)
Remitted	2901	1.8 (1.7–1.9)	1.8 (1.7–1.9)	1.8 (1.6-2.0)
No PTSD	23,213	Referent	Referent	Referent
Analysis 2^d^				
Comorbid both^e^				
Chronic PTSD	1240	7.3 (6.5–8.2)	4.7 (4.4–5.1)	9.6 (8.1–11.4)
Delayed PTSD	1270	7.3 (6.5–8.2)	4.8 (4.5–5.2)	9.1 (7.7–10.7)
Remitted PTSD	719	7.1 (6.3–8.1)	4.7 (4.3–5.1)	7.3 (6.1–8.8)
No PTSD	1265	6.3 (5.6–7.1)	4.6 (4.3–4.9)	7.1 (6.0–8.4)
Comorbid depression/anxiety only				
Chronic	227	6.4 (5.5–7.5)	5.1 (4.6–5.6)	8.8 (7.0–11.1)
Delayed	273	6.0 (5.1–7.1)	5.2 (4.8–5.7)	8.8 (7.0–11.1)
Remitted	173	4.5 (3.5–5.8)	4.3 (3.8–4.9)	5.0 (3.4–7.4)
No PTSD	538	3.9 (3.3–4.7)	4.4 (4.0-4.9)	5.1 (3.9–6.5)
Comorbid physical condition only				
Chronic	211	6.8 (5.7-8.0)	3.9 (3.4–4.4)	5.3 (3.9-7.0)
Delayed	434	6.5 (5.7–7.5)	3.8 (3.4–4.1)	5.3 (4.2–6.7)
Remitted	1478	5.6 (4.9–6.3)	3.1 (2.8–3.3)	3.3 (2.7–4.1)
No PTSD	13,070	3.4 (3.1–3.8)	1.8 (1.7–1.9)	2.2 (1.9–2.6)
Comorbid neither				
Chronic	71	4.1 (2.8–6.1)	3.5 (2.7–4.5)	3.4 (1.8–6.8)
Delayed	158	4.5 (3.3–6.1)	3.1 (2.6–3.8)	3.8 (2.4-6.0)
Remitted	531	2.6 (2.0-3.3)	2.1 (1.8–2.4)	1.8 (1.2–2.8)
No PTSD	8340	Referent	Referent	Referent

*PTSD* posttraumatic stress disorder

^a^Adjusted for sociodemographic characteristics (age, gender, race, income, employment status, physical activity, BMI, smoking status), Registry eligibility group and social support

^b^≥ 14 unhealthy or ≥ 14 activity limitation days in the last 30 days

^c^The multivariate analyses did not include physical and mental health comorbidity

^d^The multivariate analyses included physical and mental health comorbidity

^e^Comorbid both depression/anxiety at Wave 3 and any of ever reported physical health conditions

When physical and mental health comorbidity status was accounted for in Analysis 2 (Table 3), the APR increased to 7.3 for poor or fair health status (95% CI 6.5, 8.2), 4.7 for ≥ 14 unhealthy days (95% CI 4.4, 5.1), and 9.6 for ≥ 14 activity limitation days (95% CI 8.1, 11.4) in those with chronic PTSD who also had comorbid physical and mental health conditions compared to the no-PTSD group that had no comorbidity. The highest risk for poor HRQOL was observed among those with both physical and mental health conditions, followed by comorbid mental health conditions alone and comorbid physical health conditions alone; the lowest risk was found among those with no comorbidity. Similar risk estimates for poor HRQOL were also observed among those with delayed PTSD.

In conducting the sensitivity analysis, we found attenuated but significant associations between PTSD trajectory and each of the three HRQOL indicators, when accounting for physical and mental health comorbidity. The APR was 5.5 for poor or fair health status (95% CI 4.7, 6.5), 4.3 for ≥ 14 unhealthy days (95% CI 3.9, 4.8), and 8.3 for ≥ 14 activity limitation days (95% CI 6.5, 10.6) for individuals with chronic PTSD who also had comorbid physical and mental health conditions compared to the no-PTSD group that had no comorbidity (Supplementary Table 1).

## Discussion

More than a decade after 9/11, a substantial proportion of a cohort of persons exposed to the 9/11 disaster reported significant physical and mental health comorbidity and diminished HRQOL, particularly among those with PTSD. The results of this study can be best summarized through three main findings. First, the physical and mental health burden among those with PTSD is striking and worsens over the period of chronic PTSD symptoms. Second, active PTSD (chronic and delayed), regardless of the duration, was significantly associated with each of three indicators of poor HRQOL, independent of sociodemographic characteristics, social support, and comorbidity. Third, the magnitude of the effect of PTSD trajectory on each of the HRQOL indicators varied depending on the presence of physical and mental health comorbidity after adjusting for covariates.

The physical and mental health burden among 9/11-exposed individuals was significant years after exposure, particularly among those with chronic PTSD. Of numerous physical and mental health conditions examined, the age-adjusted prevalence among the entire study population was higher than in the general population for a number of conditions: depression (14.9 vs. 8.2%) [38], asthma (19.6 vs. 8.4%) [39], chronic bronchitis (8.7 vs. 4.4%) [40], and pulmonary fibrosis (0.8 vs. 0.04–0.06%) [41]. When age-adjusted prevalence of health conditions was stratified by PTSD trajectory, enrollees with chronic PTSD were 14 to 15 times more likely to have depression or anxiety at W3, and 1.4 to 6.8 times more likely to have ever reported physical health conditions (1.4 for hypertension and 6.8 times for pulmonary fibrosis) than those with no PTSD. The higher prevalence of physical and mental health conditions in this study is consistent with the existing 9/11 literature documenting the impact of exposure on adverse health outcomes.

Early data from the National Comorbidity Survey revealed that more than one-third of individuals with PTSD failed to recover, even after many years [7]. Similarly, one-third of our study population remained symptomatic over a nearly 10-year period of follow-up. Consistent with other studies [6, 19], we also found that PTSD symptoms were significantly associated with impaired HRQOL.

However, the effect of PTSD trajectory on HRQOL was largely impacted by physical or mental health comorbidities in this study. Depression/anxiety and physical health conditions amplify the adverse effect of PTSD symptoms on quality of life. Those with chronic or delayed PTSD and comorbid physical and mental health conditions experienced the greatest decrement in HRQOL, followed by those with a mental or physical health condition alone. Chronic and delayed PTSD did not appear to be differentially associated with either of the poor HRQOL indicators; this suggests that in the presence of PTSD, HRQOL may be more strongly influenced by comorbid conditions than the duration of PTSD. Notably, the comorbid depression/anxiety has a greater impact on the PTSD-HRQOL (specifically, unhealthy days and activity limitation days) associations than comorbid physical health condition only. These high-risk groups may have more severe health problems or conditions that are not being well managed or treated. Understanding and mitigating the impact of the co-occurrence of two or more health conditions on PTSD trajectory and poor HRQOL has important implications for treatment planning and future disaster preparedness and response.

One of the HRQOL impairment indicators, activity limitation, emerged as an important issue in the population studied. Frequent limited activity is a major source of health and economic burden at both the individual and societal level [42, 43]. In the present study, individuals with chronic and delayed PTSD and comorbid physical and mental health conditions experienced nine times the risk of activity limitation (≥ 14 in the last 30 days) compared to those without PTSD and comorbidity, further highlighting the debilitating effects of comorbidity. A previous study using Registry data found that co-occurring PTSD and respiratory illness was significantly associated with premature labor force exit and lower income [44]. As such, our findings may have broad public health and economic implications. It is important to understand the magnitude of comorbidity’s impact on the HRQOL of those with PTSD, and to account for multiple comorbidities when providing clinical care to patients with PTSD. This also underscores the importance of integration of physical and mental health care.

Research has shown that improvement in PTSD symptoms can result in better HRQOL [6]. In this study, improved HRQOL (reduced poor or fair health status and decreased unhealthy days) among those with remitted PTSD was observed in those with comorbid mental or physical health conditions alone and in those with neither comorbidity, but not in those with both mental and physical comorbid conditions. Thus, multiple comorbidities are an important factor in HRQOL impairment among individuals with remitted PTSD.

There are several strengths to this study. This is the first 9/11 study to assess the association between PTSD trajectory and HRQOL, and how the magnitude of the association changes in the presence of physical and/or mental health comorbidity 10–11 years after 9/11. In assessing comorbidity, this study examined numerous physical and mental health conditions, including depression, anxiety, and 18 physician-diagnosed physical health conditions. Furthermore, the study population included both first responders and survivors from the affected area, broadening the external validity of the findings. Lastly, the large post-disaster population and availability of important demographic covariates and other HRQOL risk factors offer sufficient power to examine the association between post-9/11 PTSD trajectory and HRQOL.

The findings in this study are subject to some limitations. First, our findings may be distorted by potential selection bias resulting from loss to follow-up if attrition is related to both the exposure and outcome under study [45, 46]. The results of sensitivity analysis on the associations between PTSD trajectory and HRQOL, accounting for comorbid physical and mental health conditions, were significant and in the same direction as in our complete case analysis. But the results among W3 non-participants (Supplementary Table 1) were attenuated, suggesting attrition may have influenced the results and that those lost to follow-up may be healthier than those who chose to stay in the study. A second limitation is the reliance on self-report to identify mental and physical health conditions. However, the use of validated, standardized questionnaire measures to identify mental disorders (i.e., PTSD, depression, and anxiety) and HRQOL may help to minimize potential reporting bias. Lastly, lack of clinical information on treatment (e.g., type and duration of medication) impeded our ability to control for these variables in the analysis.

## Conclusions

This study quantifies the extent to which comorbid depression, anxiety, and chronic physical conditions affect the association between 9/11-related PTSD trajectories and HRQOL. Our findings have implications for both PTSD researchers and clinicians. The substantial psychological and physical comorbidity in chronic PTSD patients and its contribution to a greater decrement of HRQOL years after the WTC exposure is a cause for longer-term public health concern. Early and repeated screening for PTSD and comorbid health conditions among those exposed to disaster or trauma is an important component of future disaster response and follow-up in order to provide services to affected individuals and an opportunity to prevent and reduce comorbidity. In short, optimizing functioning and improving HRQOL through integration of physical and mental health care at both the individual and community level may be an important target in the early identification and treatment of PTSD and comorbidity.

## Electronic supplementary material

Below is the link to the electronic supplementary material.

Supplementary material 1 (DOCX 15 KB)
