# Supplementary material for: Comorbidity amplifies the effects of post-9/11 posttraumatic stress disorder trajectories on health-related quality of life
Source: Qual Life Res. 2017 Dec 20;27(3):651–60. doi: 10.1007/s11136-017-1764-5 (PMC5845593; doi:10.1007/s11136-017-1764-5)
Supplement: Supplementary file 1 — Supplementary material 1 (DOCX 15 KB) [file 11136_2017_1764_MOESM1_ESM.docx]

**Title**: Comorbidity amplifies the effects of post-9/11 posttraumatic stress disorder trajectories on health-related quality of life

**Authors**: Jiehui Li, Kimberly Caramanica Zweig, Robert M. Brackbill, Mark R. Farfel, James E. Cone

**Affiliation**: New York City Department of Health and Mental Hygiene, World Trade Center Health Registry, New York, NY

**E-mail address of the corresponding author**: [jli3@health.nyc.gov](mailto:jli3@health.nyc.gov)

| **Supplementary Table 1**. Adjusted prevalence ratio (APR) of PTSD trajectory with poor HRQOL by physical and mental comorbidity among adult WTCHR enrollees who participated in Waves 1 and 2 but not in Wave 3 (N=8,164) | | | | | |
| --- | --- | --- | --- | --- | --- |
|  | | |  |  | |
|  |  | APR (95% Confidence Interval)^*^ | | | |
|  | N | Fair/poor general health | ≥14 Unhealthy days^**^ | ≥14 Activity limitation days^**^ | |
| *Analysis 2* |  |  |  |  | |
| Comorbid both^***^ |  |  |  |  | |
| Chronic PTSD^****^ | 412 | 5.5 (4.7-6.5) | 4.3 (3.9-4.8) | 8.3 (6.5-10.6) | |
| Delayed PTSD | 305 | 5.7 (4.8-6.8) | 4.3 (3.9-4.8) | 7.5 (5.9-9.6) | |
| Remitted PTSD | 101 | 5.0 (3.9-6.4) | 3.2 (2.7-3.9) | 4.2 (2.7-6.6) | |
| No PTSD | 657 | 3.3 (2.8-3.9) | 2.8 (2.5-3.2) | 3.3 (2.5-4.4) | |
| Comorbid depression/anxiety only |  |  |  |  | |
| Chronic | 145 | 4.3 (3.5-5.4) | 4.1 (3.6-4.7) | 7.7 (5.8-10.2) | |
| Delayed | 139 | 3.5 (2.7-4.6) | 3.6 (3.1-4.2) | 5.7 (4.1-7.9) | |
| Remitted | 68 | 2.5 (1.6-4.0) | 3.1 (2.5-3.9) | 2.7 (1.4-5.3) | |
| No PTSD | 539 | 1.5 (1.1-2.0) | 2.2 (1.9-2.6) | 2.1 (1.4-2.9) | |
| Comorbid physical condition only |  |  |  |  | |
| Chronic | 139 | 5.1 (4.2-6.3) | 4.0 (3.5-4.6) | 5.6 (4.0-7.9) | |
| Delayed | 179 | 4.8 (3.9-5.9) | 3.9 (3.4-4.4) | 7.1 (5.3-9.6) | |
| Remitted | 145 | 3.7 (2.9-4.8) | 2.4 (2.0-3.0) | 3.1 (2.0-4.8) | |
| No PTSD | 2,204 | 2.6 (2.2-3.0) | 1.6 (1.4-1.8) | 1.9 (1.5-2.5) | |
| Comorbid neither |  |  |  |  | |
| Chronic | 87 | 4.1 (3.1-5.5) | 3.7 (3.1-4.4) | 4.8 (3.1-7.4) | |
| Delayed | 157 | 3.9 (3.0-5.1) | 3.3 (2.8-3.8) | 5.3 (3.6-7.7) | |
| Remitted | 119 | 2.4 (1.6-3.5) | 2.0 (1.6-2.7) | 1.9 (0.97-3.7) | |
| No PTSD | 2,768 | Referent | Referent | Referent | |
| *PTSD* posttraumatic stress disorder. | | | | |  |
| ^*^Adjusted for sociodemographic characteristics (age on 9/11, gender, race, household income, employment and smoking status), and Registry eligibility group. | | | | |  |
| ^**^ ≥14 unhealthy or ≥14 activity limitation days in the last 30 days. | | | | |  |
| *** Comorbid both physician-diagnosed depression/anxiety and any of 11 physical health conditions reported at Wave 2. The physical health conditions included hypertension, angina, heart attack, coronary heart disease, stroke, sarcoidosis, asthma, reactive airways dysfunction syndrome, chronic bronchitis, emphysema, and diabetes. | | | | |  |
| **** Four PTSD trajectory groups based on their PTSD status across waves: chronic (W1+ and W2+), delayed (W1- and W2+), remitted (W1+ and W2-), and no  PTSD (W1- and W2-). | | | | |  |
